# Bizarre Parosteal Osteochondromatous Proliferation (Nora Lesion): A Narrative Review

**DOI:** 10.15388/Amed.2022.29.2.4

**Published:** 2022-06-29

**Authors:** Ipponi Edoardo, Ferrari Elisa, Ruinato Alfio Damiano, De Franco Silvia, Capanna Rodolfo, Andreani Lorenzo

**Affiliations:** University of Pisa: Department of Orthopedics and Trauma Surgery; University of Pisa: Department of Orthopedics and Trauma Surgery; University of Pisa: Department of Orthopedics and Trauma Surgery; University of Pisa: Department of Orthopedics and Trauma Surgery; University of Pisa: Department of Orthopedics and Trauma Surgery; University of Pisa: Department of Orthopedics and Trauma Surgery

**Keywords:** Bizarre parosteal osteochondromatous proliferation, BPOP, Nora lesion

## Abstract

**Background:**

Bizarre parosteal osteochondromatous proliferation (BPOP), or Nora lesion, is a rare proliferative disease arising from the parosteal region of bones. Although BPOP’s pathogenesis is still not certain, modern literature suggests it to be a tumor-like lesion or even a benign neoplasm. Due to the extremely low incidence, to this date studies on the topic are limited to case reports and a few case series. This narrative review aims to resume literature on BPOP and provide an overview of its natural history, morphologic characteristics and prognostic horizon.

**Materials and methods:**

A systematic research of the literature was done to identify studies reporting on patients who suffered from BPOP between 1983 and 2021. We collected data regarding aetiologic and pathogenetic theories, patients’ personal data and anamnesis, lesions’ location, clinical presentation, imaging features, pathological appearance, treatment and prognosis.

**Results:**

We identified 322 cases of BPOP with a mean age of 34.3 years at the moment of diagnosis. There was no gender difference. The most involved site was the hand, followed by the foot. A history of trauma was reported for 14.7% of the cases. 38.7% of the patients had pain. Literature defined typical radiographic and microscopic patterns that characterize Nora lesions. While imaging is fundamental to orientate towards BPOP, histological evaluation is mandatory to get the definitive diagnosis. To this date, only reliable therapeutic option is represented by surgical resection. BPOP is burdened by a risk of recurrence that accounts to 37.4%.

**Conclusion:**

BPOP is a rare benign disease that should be considered during the differential diagnosis of parosteal lesions, especially in the acral regions. Careful diagnostic evaluations are necessary to get the correct diagnosis and wide margins of resection are recommended to minimize the relatively high risk of local recurrence.

## Santrauka.

**Įvadas:**

Neįprasta parostealinė osteochondromatozės proliferacija (BPOP), arba Noros pažeidimas, yra reta proliferacinė liga, atsirandanti dėl kaulų parostealinės srities. Nors BPOP patogenezė vis dar nėra iki galo aiški, šiuolaikinėje literatūroje nurodoma, kad tai yra naviko pažeidimas ar net gerybinis navikas. Dėl labai nedidelio sergamumo iki šiol tyrimai šia tema apsiriboja sveikatos priežiūros įstaigų pranešimais ir keliais atvejais. Šia apžvalga siekiama atnaujinti BPOP literatūrą ir apžvelgti jos atsiradimo istoriją, morfologines charakteristikas ir prognozes.

**Medžiagos ir metodai:**

Atlikta sisteminga literatūros apžvalga, siekiant rasti tyrimų, kuriuose pranešama apie pacientus, 1983–2021 m. sirgusius BPOP. Surinkti duomenys apie etiologines ir patogenetines teorijas, pacientų asmens duomenis ir anamnezę, pažeidimų lokaciją, klinikinius simptomus, magnetinio rezonanso vizualizavimo ypatybes, patologinę išvaizdą, gydymą ir prognozę.

**Rezultatai:**

Nustatyti 322 BPOP atvejai, paciento vidutinis amžius diagnozės metu buvo 34,3 metų. Lyčių skirtumo nebuvo. Dažniausia pažeidimo vieta buvo ranka, po to pėda. Traumų istorija buvo būdinga 14,7 proc. visų atvejų. 38,7 proc. pacientų jautė skausmą. Literatūroje apibrėžti tipiniai radiografiniai ir mikroskopiniai modeliai, apibūdinantys Noros pažeidimus. Nors magnetinio rezonanso vaizdai yra labai svarbūs, norint nustatyti BPOP, histologinis vertinimas yra būtinas galutinei diagnozei. Iki šiol patikimiausias gydymo būdas yra tik chirurginė rezekcija. BPOP apsunkina tai, kad ligos pasikartojimo rizika sudaro 37,4 proc.

**Išvada:**

BPOP yra reta gerybinė liga, į kurią turi būti atsižvelgiama diagnozuojant diferencinius parostealinius pažeidimus, ypač galūnėse. Teisingai diagnozei gauti būtina atlikti kruopštų diagnostinį vertinimą, o norint sumažinti santykinai didelę lokalaus pasikartojimo riziką, rekomenduojama atlikti platesnę rezekciją.

## Introduction

Although a large variety of diseases can be responsible for osteochondral proliferation in the parosteal region, its onset is extremely rare in the distal segments of the human body. At the end of the 1970s, the osteochondral lesions that were most likely to present with parosteal localization were osteochondromas and parosteal chondromas. Both were only rarely found in the distal extremities, as emerged from the Mayo Clinic’s casuistry, reported by Dahlin in 1978 [1,2]. In particular, the document testified that only 14 of the 516 documented osteochondromas (2.7%) involved the bones of hands and feet. In parallel with the extremely low incidence of these tumors, through the years the same institution had hospitalized and treated dozens of cases who suffered from other parosteal osteochondromatous neoformations which mainly occurred in the distal segments of the upper and lower limbs and that could not meet the radiological and pathological criteria for the diagnosis of osteochondroma, periosteal chondroma or other pathologies then known. In 1983 Nora et al. [1] condensed this experience in an article, reporting 35 cases of lesions involving hands and feet with similar radiographic and histological patterns. In light of these similarities, and the substantial differences with other pathologies known to that date, authors proposed the introduction of a new and undescribed disease, which they named “Bizarre Parosteal Osteochondromatous Proliferation of the small bones of the hands and the feet’’. Although this first study verged only on lesions of the extremities, the authors already noted that identical lesions had also been seen to involve the long bones in two cases. This opened to the idea that the disease could also be found in proximal regions. In the years that followed, several case reports and case series [3-82] increased the number of known cases which had the same clinical, radiographic and histological characteristics described by Nora et al. [1], with a certain share of lesions involving also the proximal skeleton. In light of this evidence, the disease was definitely accepted as a separate entity and is now commonly referred to as the “Bizarre Parosteal Osteochondromatous Proliferation” or “Nora lesion”, in honor of its discoverer.

The aim of our study is to resume international literature on this topic and provide an overview of Nora lesions’ characteristics. With this aim, we resumed the data available to this date to give an overall picture of Nora lesion’s epidemiology, possible risk factors, clinical presentation, imaging and histological characteristics, but also the possible therapeutic approaches and their effects on patients’ prognosis.

## Search strategy and data management

A systematic review of the literature was carried out in order to identify in international literature studies on the Bizarre Parosteal Osteochondromatous Proliferation (BPOP), also known as Nora lesion. Our analysis was performed searching in PubMed, Scopus and Google Scholar the terms “Bizarre”, “Parosteal”, “Osteochondromatous” and “Proliferation”, as well as “BPOP” and “Nora Lesion” with all their possible combinations. The research was extended to the studies published between 1983 and December 31st, 2021. Studies in English language, with available full-text and explicitly reporting on patients with BPOP were included in this review. Exclusion criteria were represented by the use of languages other than English and the absence of a clear histological diagnosis. Both retrospective studies and prospective trials were considered. The full text of each article potentially suitable for the purpose of our analysis was viewed independently by two different authors (EI and EF). Each patients’ personal data including age at diagnosis and gender were recorded, as well as lesions’ location and size. We considered whether cases had or not a history of trauma in their lesion site. Data about clinical presentation such as pain, reduced articular mobility and functional limitations were included in our database when reported by their authors. Considerations about imaging and anatomical characteristics of Nora lesions have been analyzed to get a multi-comprehensive view of the disease. In parallel, when referred, all the surgical approaches were recorded, alongside eventual postoperative complications and clinical outcomes after surgery. All those studies which reported patients’ prognosis, in particular whether they suffered or not from local recurrence, were collected in order to assess a global recurrence-free survival rate. In general, the focus of the references varied broadly. Studies could either be case reports or case series and investigate one or more specific aspects like preoperative presentation, imaging, pathology, genetics, surgical treatments and postoperative outcomes.

**Figure 1.**
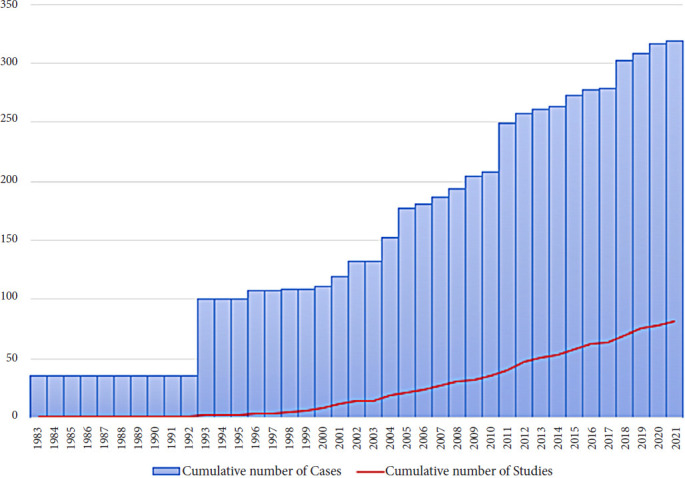
Graphic representation of cumulative number of cases and studies through the years since the discovery of the disease by Nora et al. in 1983 [1].

81 articles met our selection criteria. 55 of them were case reports, whereas the remaining 26 were case series with a population size ranging between 2 and 69 units (mean 10.3). In total, our search led to the identification of 322 documented cases reported in literature through a period of 38 years. The chronological evolution of BPOP’s overall casuistry is resumed in Figure 1.

## Epidemiology

Bizarre Parosteal Osteochondromatous Proliferation is an extremely rare disease, with less than 350 cases described in literature since its discovery in 1983. Our research led to the identification of 322 different cases with histopathological diagnosis of BPOP. 49.8% of those patients were females and 50.2% were males, testifying an equal distribution of the disease between the two genders. Patients’ mean age, calculated on 306 cases, was 34.3 years (2-81).

Nora lesions arose from the upper limb in 223 cases (69.2%). In particular, the bones of the hand were the most frequently affected, accounting alone for a total of 191 cases (59.3%). Metacarpal bones were involved in 38 cases (11.8%) [1, 3-17], proximal phalanx in 41 (12.7%) [1, 3, 7-10, 18-24], middle phalanx in 44 (13.6%) [1, 3, 6-10, 13, 19, 20, 25-29] and distal phalanx in 19 (5.9%) [1, 3, 7-10, 19, 30, 31]. The exact localization of the disease inside the hand was unknown in the other 49 patients. Nora lesions involved radius [5, 32-37] and ulna [5, 30, 33-35, 37-43], respectively, in 10 and 17 cases. 4 (1.2%) lesions were diagnosed nearby the humerus [33, 44-46] and only a single case in literature was found in the clavicular region [18].

87 patients (27.0%) developed Nora lesions in their lower limbs. Neoformation involved femur [3, 16, 33, 37, 47, 48], tibia [3, 9, 18, 33, 49, 50] and fibula [18, 33, 51] in 11 (3.4%), 8 (2.5%) and 4 cases (1.2%), respectively. One additional case was discovered nearby the patella [52]. The foot was by far the most frequently involved site in the lower limb, with 62 (19.2%) documented cases. In 17 of them (5.3%), the exact site of the lesion was not reported [33, 46, 50, 53]. Only 3 cases (0.9%) involved the hindfoot – calcaneus in 1 case [54] and talus in 2 cases [14, 55] – whereas the large majority of cases arose from the forefoot. 29 of them were localized in the metatarsal area [1, 5, 9, 16, 23, 34, 56-64], with 3 in particular arising from sesamoid bones [5, 65, 66]. Proximal phalanx was involved in 11 cases [1, 9, 67-72] and distal phalanx in 2 cases [73, 74], while no BPOP of the middle phalanx of the foot has been described with certainty in literature to this date.

8 patients suffered from Nora lesions localized in their head bones [33], including mandible (4; 1,2%) [75-78], maxilla (1; 0.3%) [79], zygoma (1; 0.3%) [80], and nose (1; 0.3%) [81]. Only one case of spine localization has been described in literature to this date [82]. Lesion’s localization was not provided for 4 cases: 3 by Nora et al. in 1983 and for 1 by Cocks et al. in 2018 [1, 7].

**Figure 2.**
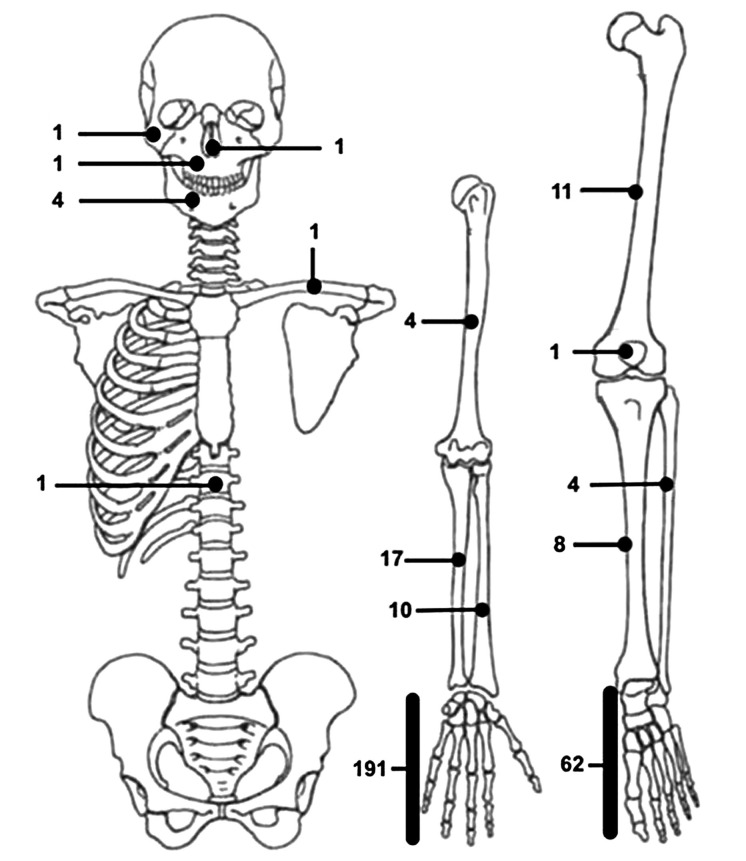
Schematic distribution of Nora lesions included in our literature review.

The distribution of Nora lesions in terms of locations and frequency is graphically represented in Figure 2, while a schematic overview in detail is reported in Table 1.

**Table 1.** Detailed distribution of all cases of BPOP included in our review. Each localization (left column) was associated with its overall number of cases (right column).

Localization	Cases (n)
**HAND**	**191**
Metacarpal bones	38
Proximal phalanx	41
Middle phalanx	44
Distal phalanx	19
Unknown	49
**RADIUS**	**10**
Distal	1
Midshaft	3
Proximal	1
Unknown	5
**ULNA**	**17**
Distal	6
Midshaft	3
Unknown	8
**HUMERUS**	**4**
Distal	1
Midshaft	1
Proximal	1
Unknown	1
**CLAVICLE**	**1**
**HEAD**	**8**
Nose	1
Zygoma	1
Maxilla	1
Mandible	4
Unknown	1
**SPINE**	**1**
**FEMUR**	**11**
Distal	6
Unknown	5
**PATELLA**	**1**
**TIBIA**	**8**
Proximal	4
Unknown	4
**FIBULA**	**4**
Distal	2
Unknown	2
**FOOT**	**62**
Calcaneus	1
Talus	2
Metatarsal bones	26
Sesamoid bones	3
Proximal phalanx	11
Distal phalanx	2
Unknown	3
**UNSPECIFIED**	**4**

## Etiology and pathogenesis

To this date, BPOP’s etiology and pathogenesis still remain controversial. For years, several authors hypothesized it could be a reactive neoformation consequential to a traumatic event. Post-traumatic calcifications of the soft tissues are largely described in literature, and periosteum, as well as the other connective tissues adjacent to it, could theoretically calcify as a result of a reparative process that follows deep hematomas or localized flogistic events. Nora lesions have radiographic and histologic characteristics that remind of the ossification which typically follows a reparative process of periosteum or growing plates’ cartilage after a traumatic lesion [1, 33].

Among the 217 cases with detailed anamnestic and clinical presentation described in literature, 32 had a trauma in the location which later hosted the Nora lesion. Cases with a history of trauma therefore account for a total of 14.7% of the cases, whereas the remaining 85.3% did not suffer from significant traumatic events (Table 2).

**Table 2.** Synthetic resume of the findings obtained by studies with 5 or more cases. The others were gathered in a single line, under the name “Other studies”.

Study	Cases	Mean Age	Gender		Trauma		Clinical presentation		Local recurrence
**Name**	**N**	**Years**	**F**	**M**	**Yes**	**No**	**Painful**	**Painless**	**%**
Abramovici et al., 2002	12	30.3 (12-63)	8	4	1	11	4	8	17%
Berber et al., 2011	22	31.8 (6-66)	8	14	-	-	8	9	27%
Cocks et al., 2018	16	(18-79)	5	11	-	-	-	-	-
Dhondt et al., 2005	24	38.8 (12-81)	9	15	-	-	-	-	29%
Jibu et al., 2011	13	40.1 (13-65)	7	6	2	11	5	8	54%
Meneses et al., 1993	65	33.9 (8-73)	34	31	9	56	3	27	55%
Michelsen et al., 2004	10	41.7 (23-63)	6	4	1	9	2	8	22%
Nilsson et al., 2004	5	36.8 (24-46)	3	2	-	-	-	-	40%
Nora et al., 1983	35	34 (14-74)	19	16	0	35	5	13	51%
Teoh et al., 2009	11	37 (14-65)	7	4	1	10	2	9	63%
Kalem et al., 2015	6	39 (17-62)	4	2	2	4	4	2	50%
Smith et al., 1996	7	30 (18-37)	1	6	5	2	4	3	43%
Other cases	96	34.0 (3-64)	48	45	11	83	47	45	26%

When evaluating these data, we must consider that the distribution of cases with a history of trauma varies largely between one study and the other. For example, Nora et al. [1] excluded any history of trauma for their 36 cases, whereas injuries were present in the anamnesis of 5 out of 7 patients in the study published by Smith et al. in 1996 [46]. This difference could be attributable to diverse standards used by the authors to define a traumatic event as potentially relevant. Contextually, the entity of the injury, as well as the time between its occurrence and the definitive diagnosis of disease, were only rarely reported in literature. In light of the low incidence of the disease compared to the high frequency of traumas, it is unlikely that localized injuries alone may represent the etiologic cause of BPOP’s outbreak. On the other hand, they could play a role in a multifactorial pathogenesis, representing a risk factor for already predisposed patients.

In parallel with the post-traumatic hypothesis, since its discovery by Nora et al. the lesion has been speculated to be a tumor-like lesion or even a neoplasm. This theory has always been supported by the extremely high risk of local recurrences testified by several authors and in part by the histologic appearance of the lesions, described below in a dedicated paragraph [1, 3, 5, 6, 8-10, 17, 18, 23, 28-31, 33-37, 39, 46, 50, 53, 70-76]. Although in the first moment some authors theorized that BPOP could have been an intermediate stage that connected florid reactive periostitis with Turret exostosis [83, 84], further studies definitely established Nora lesion as an independent pathological entity [8, 9, 22 33]. Some studies also focused on the genetic characterization of BPOP, in order to allow a better comprehension of lesions’ nature. In 2004, using chromosome banding and fluorescence in situ hybridization (FISH) analyses, Nillson et al. [53] detected a balanced translocation between chromosome 1 and chromosome 17: t (1;17) (q32;q21). The same mutation was found in the same year by Zambrano et al., in their only case who had a local recurrence after surgical excision [35]. A similar translocation between the two chromosomes, t(1;17)(q 42;q23), was discovered by Endo et al. [68] only one year later, in 2005. In both cases translocations involved several genes, including BRCA1, an oncosuppressor gene related among the others with breast carcinoma, and COL1A1, involved in the deposition of type 1 collagen whose disregulations have been demonstrated to be associated with diseases such as dermatofibrosarcoma protuberans and giant cell fibroblastoma. Furthermore, in 2021 Zhang et al. [85] discovered a correlation between the disruption of the Jmjd3/p16 Ink4a signaling pathway and the onset of BPOP in a mice model. These evidences reinforce the idea that BPOP should be considered as a benign neoplasm, although the influence of these genes on the outbreak of the disease is still uncertain and the full knowledge of its etiology and pathological pathways are still far from being fully known [19].

## Clinical presentation

BPOP can either stay asymptomatic for a long period of time and be diagnosed as an incidental lesion or overcome the clinical horizon leading to the onset of significant signs and symptoms. Typical symptoms – when present – include pain, palpable swelling and functional impairment. Taking into consideration all the 217 cases in literature whose clinical picture was described, the 38.7% (84) of them had localized soreness at the moment of their diagnosis (Table 2) [1, 3-6, 8-10, 12-14, 17-22, 27, 30, 31, 33-37, 40, 44, 46-50, 54, 57, 61, 62, 73, 74]. The remaining 61.3% (133) did not suffer from any pain directly attributable to the disease.

Pain apart, BPOP can also be discovered due to the progressive appearance of a swelling in the hosting region. In general, the tumefaction is described as a lone hard mass, not mobile against the underlying bony surface. Lesion’s growth is often slow but unceasing for months and even years, although cases whose size remained steady or even decreased have been recently described in literature [56]. In case these masses are not promptly diagnosed or treated, they can reach even considerable dimensions [18, 20, 44, 61-63]. Furthermore, the bigger lesions get, the higher is the risk their volumetric spread translates into visible deformations and functional limitations. Lesions that involve superficial bones or anatomical regions nearby mobile articulations are more likely to develop these consequences of the lesion’s mass effect. In particular, Nora lesions have a higher incidence on the upper limb, where several authors testified a mild-to-severe functional impairment to the fine and precise articulations of wrist, hand and fingers [3-5, 9, 14, 18-20, 26]. Similar appearances can be found in cases whose lesions are localized in the lower limb, with plantar lesions in particular that may represent an obstacle for patients’ correct load distribution and deambulation [5, 27, 54, 61]. Both Rottler et al. [55] and Reddy et al. [14] associated BPOP with local fasciitis, and it was considered by the authors whether the two diseases are comorbidities or alternatives in a differential diagnosis.

No significant correlation with systemic diseases could be observed on patients who suffered from BPOP, although the patient’s global clinical picture was portrayed only for a low number of cases. In their case report, Orui et al. [12] looked into their patient’s blood exams, identifying neutrophilic leukocytosis and increased values of alkaline phosphatase. Rottler et al. [55], for their part, documented a case with slightly increased CRP values. However, single cases are insufficient to establish whether there was a cause and effect relationship between these values and BPOP. Studies on larger populations should therefore be taken in order to get a better comprehension on the topic.

## Appearance and diagnosis

Depending on the entity of their clinical presentation, Nora lesions can come to medical attention due to the onset of the aforementioned symptoms or present as an occasional finding during a radiographic exam taken for other diagnostic purposes.

As a rule, a diagnostic approach is carried out in a two-step process. In a first step, the morphology of the mass is investigated using X-ray images. In case the radiographic findings are consistent with the ones of a Nora lesion, further multiplanar exams such as CT scan and MRI should be taken in order to achieve a better characterization of the mass. Cases with suggestive radiographic presentation require a second step, since biopsy and consequential histological examination are mandatory in order to get the definitive diagnosis of Bizarre Parosteal Osteochondromatous Proliferation.

### Imaging

Although the final diagnosis of BPOP is classically made through pathological investigation, imaging tests play a pivotal role in orienting the diagnostic process towards the disease. X-rays, but also MRI and CT scans can be useful to characterize a suspect lesion, supporting or excluding a presumptive diagnosis of Nora lesion.

*X-RAYS* – X-rays represent the first-line imaging test for bone and calcified tissues. Radiographically, BPOP presents as a well-marginated ossified mass that directly arises from the cortical surface of the underlying bone [10, 19, 63]. The lesion generally has a large implant base above an intact cortical surface. Cortical erosion has been occasionally reported, but does not represent a typical feature [19, 32].

**Figure 3.**
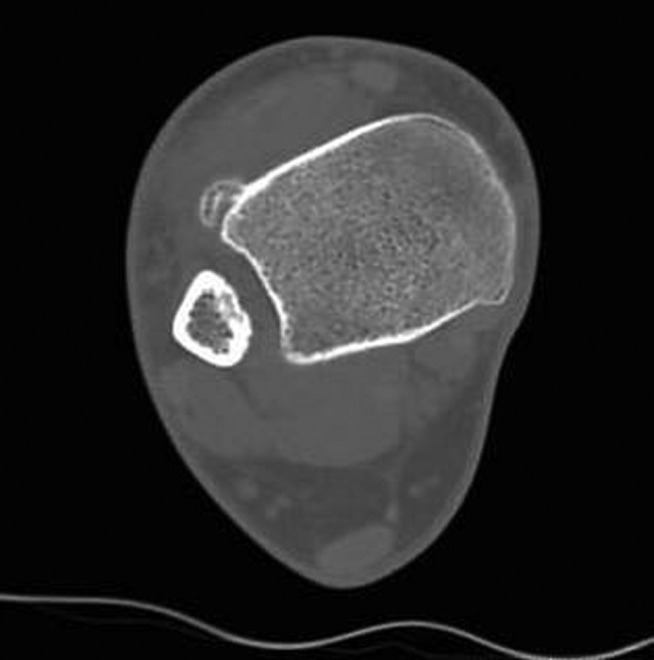
CT image of a Nora lesion arising from the antero-lateral surface of the tibia.

*CT SCANS* - CT scans provide a tridimensional radiographic view that confirms the characteristics highlighted by X-rays, adding further information about the surrounding soft tissues and the relation between the lesion and the underlying bone. CT confirms the calcific nature of the mass and excludes signs of continuity with the medullary canal or interruptions of the cortex. Moreover, it gives a better picture of the nearby soft tissues, evidencing in particular the absence of periosteal irritative reactions (Fig. 3) [3, 10, 19, 64, 67].

**Figure 4.**
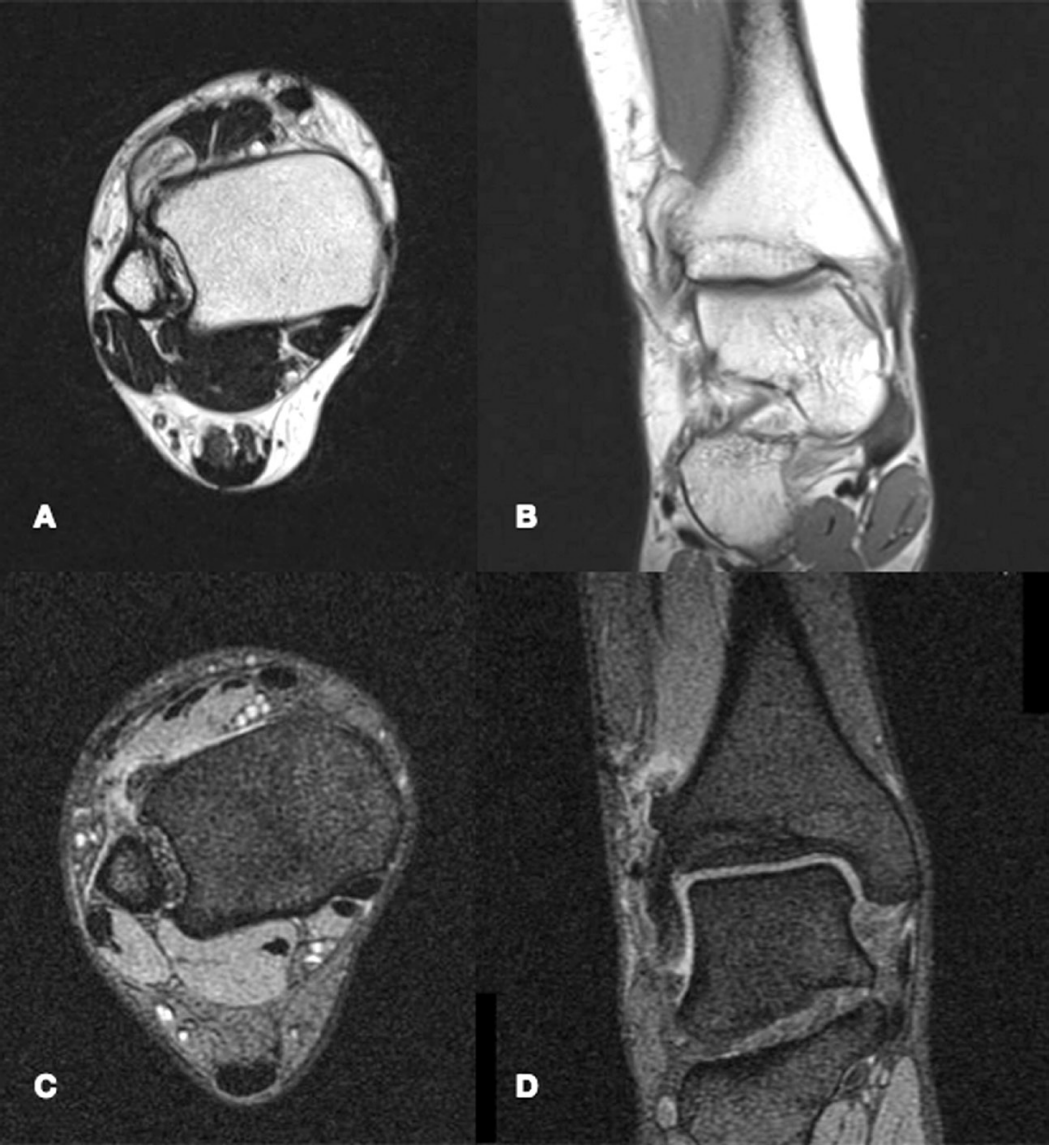
MRI images of the same lesion already pictured in Figure CT, displayed both with T1 weighted (**A** and **B**) and T2 weighted (**C** and **D**) sequences.

*MRI* – Magnetic resonance displays a Nora lesion with homogeneous low-signal intensity in T1 weighted sequences. In T2 weighted and STIR sequences, instead, the mass shows a slightly increased signal intensity in its center, with its periphery being of higher signal intensity. On T2 weighted gradient imaging, the central portion of the lesion was of inhomogeneous intermediate signal with a uniformly high signal periphery [19, 23]. Signs of periosteal reaction or perilesional oedema are exceptionally rare, although occasionally described in literature [5], and their recognition should orient physicians towards a different diagnosis (Fig. 4).

### Macroscopic and microscopic appearance

Histological examination is necessary in order to complete the diagnostic evaluation and get a definitive diagnosis of Bizarre Parosteal Osteochondromatous Proliferation.

From a macroscopic point of view, Nora lesions generally present as single or multilobulated solid bony masses, covered by a superficial cartilage cap and often surrounded by a reactive pseudocapsule that separates the neoformation itself from the surrounding soft tissues. In the operative theater, masses are commonly demarcable with ease from the overlying soft tissues, while they tend to stay firm to the periosteum. Once lesions are exposed and isolated, they generally have a bluish appearance [30, 33, 60]. Their surfaces are smooth and plain in most of the cases, although occasional sharp or irregular surfaces have been described in literature [60]. The main diameter of Nora lesions ranged between 4 and 100 millimeters in literature [1, 18, 33].

At first glance, according to several authors, this gross appearance may not significantly differ from the one of osteochondromas [1, 9, 33]. However, unlike the latter, Nora lesions have a structurally normal bone surface, without any cortical flaring [60]. Furthermore, theoretically there should be no continuity between the lesion itself and the medullary cavity of the host bone [30, 33], although cases of continuity have been described in literature [34].

From a microscopical point of view, BPOP consists of three different tissue components, expressed in different amounts: cartilage, trabecular bone and fibrous tissue [7, 33]. The lesions’ cap rarely exceeds the centimeter in thickness and is usually formed by hypercellular hyaline cartilage with signs of marked proliferative activity [3]. Chondrocytes are often large, with a bizarre shape and a certain share of them can be double-nucleated. Visible signs of tissue hypermetabolism overcome the ones of peripheral osteochondromas, almost mimicking the cellular characteristics of low-grade chondrosarcomas [1]. Inside the lesion, the osteocartilaginous interface is often irregular and a definite maturation pattern from cartilage to bone tissue is uneasy to identify, similarly to what can be found in a callus [1]. As found by Meneses et al. and later confirmed by other studies, the subchondral area is composed of fibrovascular tissue, with a form of calcified cartilage and gradual signs of endochondral ossification [1, 3, 33]. There the calcified matrix forms an irregular trabecular web that stains blue on haematoxylin–eosin coloration, a characteristic also known as “blue bone appearance” [30, 33, 34, 60]. Blue bone cellularity can range from hypercellular to hypocellular and matrix-rich [7].

Bony trabeculae are separated by a fibrous stroma with benign, reactive-looking spindle-shaped cells [3]. Focally, spindle cells round up to form osteoblasts, from which trabeculae seem to arise [33]. Mitotic figures are frequent in each cellular line, testifying the high metabolic activity of the tissues that compose the mass, while cytological atypia is infrequent [33].

### Differential diagnoses

The diagnosis of Bizarre Parosteal Osteochondromatous Proliferation is uneasy to establish due to its extremely low incidence and its paucity of pathognomonic characteristics both from the clinical and the radiographic point of view. For these reasons, the diagnostic flow often comes through a differential diagnosis with other more common diseases.

Below are listed some of the lesions that more frequently come in a differential diagnosis with BPOP. Each one is provided with a short resume that contains anamnestic, radiographic and histologic features which can direct toward the most correct diagnosis.

*OSTEOCHONDROMA* - Osteochondroma is the most common benign bone tumor in the human being. It is more likely to arise from the metaphysis of long bones and involves the hands in less than 5% of the cases. This tendency is contrary to the one of Nora lesions. In fact, hands are the most suitable location for the development of a BPOP, which hardly involves long bones and, when it does, it does not seem to prefer metaphysis over the other segments of long bones [19]. Radiographically osteochondromas appear as sessile bony neoformations surrounded by a cartilage cup. Osteochondroma’s bony roots are continuous with the original cortical bone and also the medullary cavity shows continuity within the lesion. The eventual absence of medullary continuity or the integrity of the cortical bone underlying the lesions, instead, represent key radiological findings in BPOP. Nora lesion and osteochondroma also differ in some histologic features. At microscopic examination, the cartilage cap of osteochondromas resembles a growth plate, with columns or clusters of chondrocytes evenly distributed and undergoing a maturing process which cannot be found in BPOP [36]. Furthermore, unlike Nora lesions, osteochondroma’s chondrocytes lack atypia and often are arranged in parallel lacunar spaces. Moreover, in its bony parts, osteochondroma has a regular arrangement of bone trabeculae, which are oriented at ninety degrees to the cartilage and are not irregular and puzzled like the ones present in BPOP [10, 19, 33].

*MYOSITIS OSSIFICANS* - Myositis ossificans is a benign extraskeletal ossification that occurs within muscular tissue, commonly as a consequence of a trauma. From an histological point of view it presents with three different concentric zones: a center of immature fibroblasts in a myxoid stroma with an inflammatory infiltrate, an intermediate vascularized zone with osteoid components and, finally, a rim of lamellar bone set at the edge of the mass [36, 67]. Unlike BPOP, myositis ossificans are only rarely located in the distal segments of the human body. Another difference with BPOP can be found with imaging examinations. X-rays, CT scans and MRI images taken of a myositis ossificans show a characteristic ossification that proceeds from the margins inwards [19]. This tendency is diametrically opposed to the one of BPOP, which shows an expansive evolution that extends from the center to the surrounding areas. Furthermore, in contrast to what can be found in Nora lesions which are juxtaposed to the underlying bone, myositis ossificans are usually separated from the adjacent bone and a periosteal reaction can often be identified [36, 67].

*FLORID PERIOSTITIS* - Florid reactive periostitis is a rare benign lesion that presents as an aggressive periosteal reaction associated with soft tissue swelling. It is often related to trauma and more frequently affects the small bones of the hands and feet in the second and third decade of life. Radiographically, it presents as an irregular and partially calcified mass situated peripherally to cortical bone. Microscopically, the lesion has an osteoid appearance with focally prominent surrounding osteoblasts. Florid reactive periostitis is often associated with periosteal reaction, which is not a common finding in BPOP [19, 84, 85]. Although florid periostitis and BPOP share the same locations and their radiographic appearance may often come to be similar, a careful histologic evaluation is generally able to distinguish one from the other. Microscopic evaluation also marks a difference between florid periostitis and myositis ossificans: in the first one bone tissue matures from the periphery of the lesion inwards, whereas in the latter the bone formation mainly involves the center [19, 36].

*PERIOSTEAL CHONDROMA* - Periosteal chondromas are benign tumors made of cartilage that arise in a juxtacortical position. They mainly involve metaphyseal regions, in contrast with BPOP that does not show any significant predilection. Under microscopical evaluation, juxtacortical chondromas show a majority of fibro-cartilagineous tissue with scattered matrix calcifications. This finding is similar to what can be seen in the early stages of BPOP. However, despite some similarities, periosteal chondroma is associated with cortical scalloping, cortical irregularities and periosteal reaction which are not common in BPOP [86].

*PAROSTEAL OSTEOSARCOMA* - Parosteal osteosarcoma is a low-grade malignant bone tumor that originates from the bone cortex surface. Although it prefers long bones, a short minority of cases can be found to involve the extremities and therefore enter the differential diagnosis with Nora lesions. Macroscopically, parosteal osteosarcoma appears as a dense lobulated mass with heavy mineralization and a sclerotic appearance, attached by a broad-based pedicle to the cortex [23]. On radiographs, these tumors present as densely mineralized masses arising from the bone surface. The underlying cortex is usually either normal or thickened and rarely loses its continuity. A thin radiolucent zone between the tumor and the underlying bone can be seen in the majority of cases. The imaging features that differentiate BPOP and parosteal osteosarcoma are the periosteal reaction and the infiltration of the nearby tissues: two features that are typical in osteosarcoma but absent in BPOP [36, 44]. Microscopically, parosteal osteosarcoma is a well-differentiated fibro-osseous neoplasm composed of regularly arranged bony trabeculae or broad seams of osteoid with a hypocellular spindle cell proliferation. While a low percentage of cartilage may be present, its spread is extremely limited and it lacks cellularity and cellular atypia compared to Nora lesions [19, 36].

*PAROSTEAL CHONDROSARCOMA* - Periosteal chondrosarcoma is a rare, low-grade malignant tumor mainly composed of cartilage which arises from the external surface of the bone. Peripheral chondrosarcoma, typically found in adults, is rare in the hands and feet. Radiographically, it appears as a mass with characteristic popcorn-like calcifications [67]. In order to establish a differential diagnosis between BPOP and chondrosarcoma, it is important to consider that the extremities, the most suitable locations for the development of a BPOP, are only an exceptional location for parosteal chondrosarcomas. These latter can be distinguished histologically by the well-differentiated hyaline cartilage with lobular architecture and no mitosis (in grade I) or increased cellularity and foci of necrosis (in grade II variant) [87].

## Treatment and prognosis

In light of their benign nature, Nora lesions do not require any treatment if they are small and – in particular – asymptomatic. Conversely, a treatment becomes necessary in cases when lesions imply pain and functional limitations. Several surgical approaches have been described in literature so far. Intralesional excisions, curettage and extralesional excisions have been used to treat the lesion[5, 18, 22, 32, 47].

Although the surgical approach should always depend on each patient’s necessities and each lesion’s location and size, the treatment of choice for Nora lesions is represented by en-bloc resection of the lesion as a whole, possibly with wide margins. In the vast majority of cases in literature a proper resection could be carried out without significant sacrifice of the nearby bone and soft tissues, so that no further reconstruction was necessary [5, 18, 22, 32]. For one patient, present in Barjwa et al.’s case series [18], reinforcement with a plate and crews was necessary in order to increase postoperative stability, since the mass resection caused a significant gap of the cortical bone as well. Berber et al. [5], for their part, did not mention reconstructions after their “shark bite” resections.

**Figure 5.**
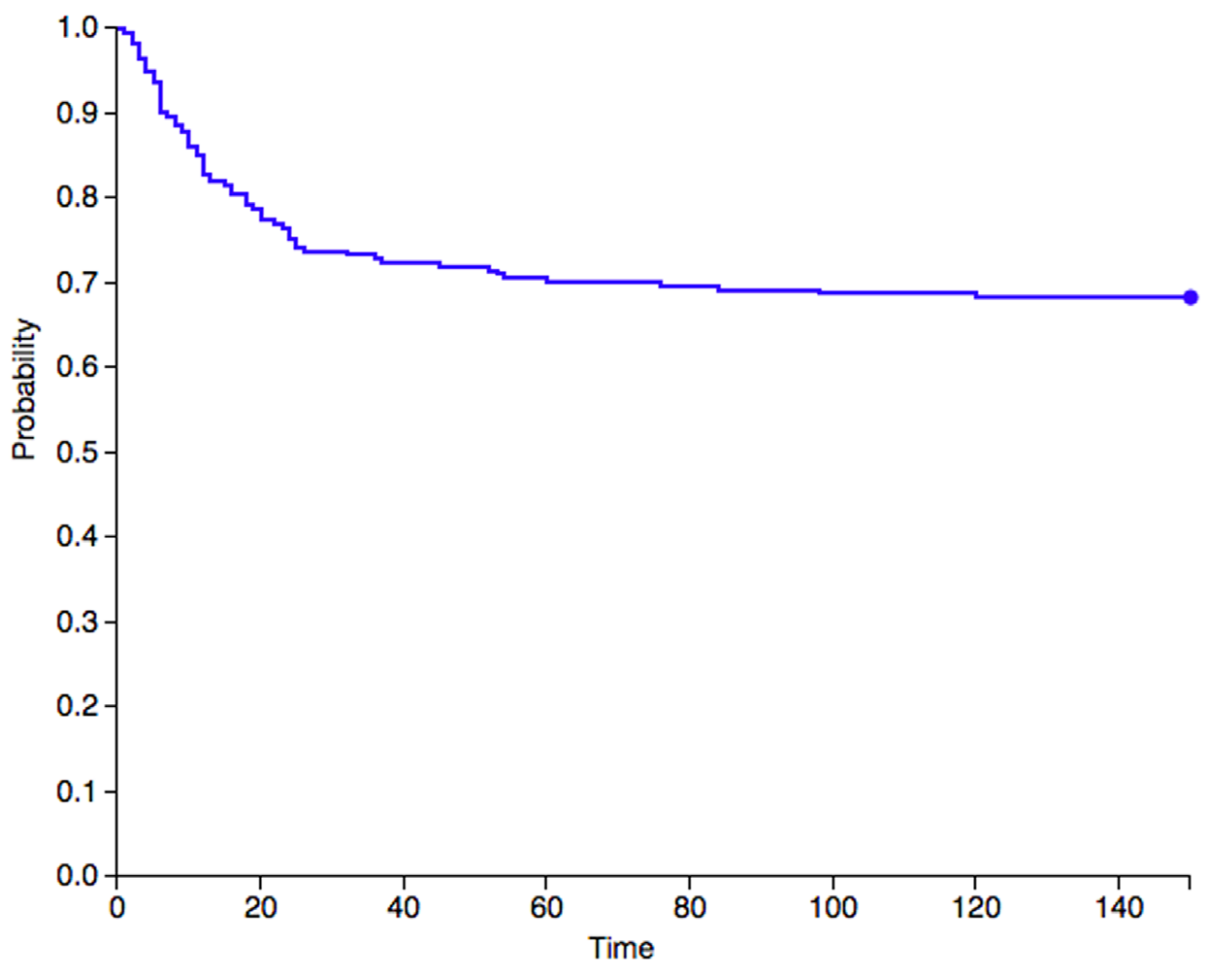
A Kaplan–Meier graph that describes the survival rates to local recurrence for Nora lesions. The graph only takes into consideration data from studies that indicate the time gap between surgery and the diagnosis of recurrence. Therefore, only 217 out of 273 cases were included.

In case lesion’s resection implies a significant bone loss, bone stock can be restored using bone grafts or prostheses. Although the use of prosthetic implants is not excluded by modern literature, with Barjwa et al. [18] who used an elbow megaprosthesis to fulfill a large bone gap left after resection, grafts represent the most suitable reconstructive approach for the majority of cases. Some authors already testified the use of both allografts and allografts [5, 11, 32, 33]. The first allows to replace or reinforce local bone with tissue of desired size and shape without the need to resect another patient’s bone. The latter, such as vascularized fibulas, provide an already vital implant to the receiving region at the cost of a sacrifice in a donor site. In particular free vascularized bone grafts, which can be associated with a soft tissue cover, can be useful when surgeons are called to face not only a lack in bone stock, but also a limited amount of overlying soft tissues that could hinder a proper wound closure. Amputation of toes, fingers or hand rays has to be reserved for exceptional cases, when the growth of wide masses in narrow anatomical segments compromised the surrounding soft tissues, precluding both an adequate wound closure and a sufficient functionality of what remains distally to the involved site [1, 4, 5, 21]. Regardless of the surgery of choice, a wide resection is mandatory in order to achieve free-of-disease margins and therefore minimize the risk of local recurrence. In fact, since its discovery by Nora et al. [1], BPOP has always been observed to be associated with significantly high rates of local recurrence after surgical treatment. Our literature analysis shows that a local recurrence was developed in 102 of the 273 cases where post-operative intercourse was described. These data highlight an overall incidence that amounts to 37.4%. The diagnosis of local secondary lesions was made between 1 month and 10 years after surgery (Figure 5). No case of metastatic lesion has been described to this date, reinforcing the idea that BPOP is to be considered effectively as a benign disease.

Our data confirm the burden of frequent recurrences in Nora lesions, which still represent a challenge for orthopedic surgeons both inside and outside the surgical theater. In today’s common practice, the achievement of a complete resection is the only intra-operative factor that could play a role in reducing recurrence rates months or years after the treatment. As far as we know, the use of systemic treatments or radiant therapy have not been experimented yet, not only due to the benign nature of the disease, but also because of our limited knowledge on BPOP’s etiology and pathogenesis. A better comprehension of the disease’s nature could provide us with new tools to fight the disease in a more complete and effective way in the near future. Coming to know the mechanisms that regulate Nora lesions’ onset and growth could allow us to make early diagnosis and perform quicker interventions with surgery and potentially even systemic or locally micro-invasive adjuvant or neoadjuvant treatments.

## Conclusion

Bizarre Parosteal Osteochondromatous Proliferation (BPOP), also known as Nora lesion, is an extremely rare parosteal disease characterized by an exophytic outgrowth from the cortical surface of the bone consisting of a peculiar combination of cartilage, osteoid and fibrous tissue. Due to both the low incidence of the disease and absence of clinical or radiographic pathognomonic signs, diagnosis is challenging even for the most experienced practitioners. The radiographic presentation of Nora lesions generally puts them in a differential diagnosis with other calcifying lesions of benign and malignant nature. Definitive diagnosis can only be made after a biopsy, under an accurate histological evaluation. Surgical resection with wide margins represents the treatment of choice, since BPOP shows a remarked tendency to give local recurrence after therapeutic approach.

## References

[1] Nora FE, Dahlin DC, Beabout JW. Bizarre parosteal osteochondromatous proliferations of the hands and feet. The American Journal of Surgical Pathology. 1983 Apr;7(3):245-250. DOI: 10.1097/00000478-198304000-00003 6837834

[2] Dahlin DC. Bone Tumors: General Aspects and Data on 6,221 cases (3rd ed.). Charles C Thomas, Sprinfield, Ill. 1978

[3] Abramovici L, Steiner GC. Bizarre parosteal osteochondromatous proliferation (Nora’s lesion): a retrospective study of 12 cases, 2 arising in long bones. Hum Pathol. 2002 Dec;33(12):1205-1210. DOI: 10.1053/hupa.2002.130103. PMID: . 12514790

[4] Barrera-Ochoa S, Lluch A, Gargallo-Margarit A, Pérez M, Vélez R. Bizarre Parosteal Osteochondromatous Proliferation (Nora’s Lesion) of the Hand: A Report of Two Atypical Cases. Case Rep Med. 2012;2012:453560. DOI: 10.1155/2012/453560. 23326274 PMC3541688

[5] Berber O, Dawson-Bowling S, Jalgaonkar A, et al. Bizarre parosteal osteochondromatous proliferation of bone clinical management of a series of 22 cases. *J Bone Joint Surg Br* 2011;93:1118–1121. DOI: 10.1302/0301-620X.93B8.26349 21768639

[6] Chamberlain AM, Anderson KL, Hoch B, Trumble TE, Weisstein JS. Benign parosteal osteochondromatous proliferation of the hand originally diagnosed as osteochondroma: a report of two cases and review. *Hand (N Y)*. 2010;5(1):106-110. DOI:10.1007/s11552-009-9217-4 19669236 PMC2820629

[7] Cocks M, Helmke E, Meyers CA, Fayad L, McCarthy E, James AW. Bizarre parosteal osteochondromatous proliferation: 16 Cases with a focus on histologic variability. *J Orthop.* 2018;15(1):138-142. Published 2018 Jan 31. DOI:10.1016/j.jor.2018.01.028 29657458 PMC5895904

[8] Dhondt E, Oudenhoven L, Khan S, Kroon HM, Hogendoorn PC, Nieborg A, Bloem JL, De Schepper A. Nora’s lesion, a distinct radiological entity? Skeletal Radiol. 2006 Jul;35(7):497-502. DOI: 10.1007/s00256-005-0041-9. Epub 2006 Apr 7. PMID: . 16602017

[9] Joseph J, Ritchie D, MacDuff E, Mahendra A. Bizarre parosteal osteochondromatous proliferation: a locally aggressive benign tumor. *Clin Orthop Relat Res.* 2011;469(7):2019-2027. DOI:10.1007/s11999-011-1898-7 21533526 PMC3111785

[10] Michelsen H, Abramovici L, Steiner G, Posner MA. Bizarre parosteal osteochondromatous proliferation (Nora’s lesion) in the hand. J Hand Surg Am. 2004 May;29(3):520-525. DOI: 10.1016/j.jhsa.2004.02.002. PMID: . 15140499

[11] Khatri K, Tiwari V, Khan SA, Nath D, Mridha AR, Raje A. Nora’s lesion of 2nd metacarpal treated by wide excision, autologous fibular grafting and metacarpophalangeal joint replacement: A rare case report. *J Clin Orthop Trauma*. 2018;9(Suppl 2):S58-S62. DOI:10.1016/j.jcot.2017.08.014 PMC6008605 29928108

[12] Orui H, Ishikawa A, Tsuchiya T, Ogino T. Magnetic resonance imaging characteristics of bizarre parosteal osteochondromatous proliferation of the hand: a case report. J Hand Surg Am. 2002 Nov;27(6):1104-1108. DOI: 10.1053/jhsu.2002.36526. PMID: . 12457364

[13] Rampoldi M, Mariano P, Casareale P. Bizarre parosteal osteochondromatous proliferation (Nora’s lesion): report of two cases. *J Orthopaed Traumatol.* 2005. 6, 101–104. DOI:10.1007/s10195-005-0091-8

[14] Reddy MV, Kandukuri A, Chandankere V, Joseph VM, Reddy AVG. Bizarre Parosteal Osteochondromatous Proliferation (Nora Lesion) in Upper and Lower Limbs: A Report of Three Cases and Review of Literature. J Orthop Case Rep. 2021 Feb;11(2):24-28. DOI: 10.13107/jocr.2021.v11.i02.2010. PMID: . 34141664 PMC8180324

[15] Ilias LM, Mohammed BA, R PS, Ponniah A, Vijayan P. Lesion with blue bone-a case report. J Pathol Nep [Internet]. 2018 Apr. 3 ;8(1):1323-1325. DOI: 10.3126/jpn.v8i1.19464

[16] Kelem M, Sahin E, Basarir K, Yildiz Y, Saglik Y. Nora’s disease: a series of six cases. Eur Res J 2015;1(3):141-145. DOI: 10.18621/eurj.2015.1.3.141

[17] Zhang Z, Purgina B, Zhang J. Bizarre Parosteal Osteochondromatous Proliferation (Nora Lesion): A Case Report and Discussion of Management. *Plastic Surgery Case Studies*. 2017 DOI: 10.1177/2513826X17751094.

[18] Bajwa SN, Reddy R, Wagh YS, Agarwal M, Katariya A. Bizarre Parosteal Osteochondromatous Proliferation- A Case Series of Typical and Atypical Presentations. J Orthop Case Rep. 2019;10(1):45-50. DOI: 10.13107/jocr.2019.v10.i01.1630. PMID: . 32547977 PMC7276577

[19] Chaabane S, Chelli Bouaziz M, Ben Ghars KH, Abid L, Jaafoura MH, Ladeb MF. Bizarre Parosteal Osteochondromatous Proliferation: Nora’s Lesion. Iran J Radiol. 2011 Sep;8(2):119-125. Epub 2011 Sep 25. PMID: ; PMCID: . 23329928 PMC3522321

[20] Flint JH, McKay PL. Bizarre parosteal osteochondromatous proliferation and periosteal chondroma: a comparative report and review of the literature. J Hand Surg Am. 2007 Jul-Aug;32(6):893-898. DOI: 10.1016/j.jhsa.2007.04.004. PMID: 17606073

[21] Gursel E, Jarrahnejad P, Arneja JS, Malamet M, Akinfolarin J, Chang YJ. Nora’s lesion: Case report and literature review of a bizarre parosteal osteochondromatous proliferation of a small finger. Can J Plast Surg. 2008 Winter;16(4):232-235. DOI: 10.1177/229255030801600406. PMID: ; PMCID: . 19949505 PMC2691031

[22] Sundaram, M., Wang, L., Rotman, M. et al. Florid reactive periostitis and bizarre parosteal osteochondromatous proliferation: pre-biopsy imaging evolution, treatment and outcome. *Skeletal Radiol* 30, 192–198 (2001). DOI: 10.1007/s002560100343 11392292

[23] Torreggiani WC, Munk PL, Al-Ismail K, O’Connell JX, Nicolaou S, Lee MJ, Masri BA. MR imaging features of bizarre parosteal osteochondromatous proliferation of bone (Nora’s lesion). Eur J Radiol. 2001 Dec;40(3):224-231. DOI: 10.1016/s0720-048x(01)00362-x. PMID: . 11731211

[24] Wang CY., Shih YJ, Chou CY, Wang CH, Chang CK, Chen TM et al. Bizarre parosteal osteochondromatous proliferation on a phalanx with periosteal erosion. 2016 *Formosan Journal of Surgery*, 49(1), 31-34. DOI: 10.1016/j.fjs.2015.07.006

[25] Martínez Álvarez S, Azorín Cuadrillero DL, Little KJ. Bizarre Parosteal Osteochondromatous Proliferation (Nora Lesion) in Pediatric Phalanges. J Hand Surg Am. 2021 Apr;46(4):344.e1-344.e9. DOI: 10.1016/j.jhsa.2020.05.002. Epub 2020 Jun 28. DOI: 10.1016/j.jhsa.2020.05.002 32611484

[26] Kumar A, Khan SA, Sampath Kumar V, Sharma MC. Bizarre parosteal osteochondromatous proliferation (Nora’s lesion) of phalanx in a child. BMJ Case Rep. 2014 Jan 23;2014:bcr2013201714. DOI: 10.1136/bcr-2013-201714. PMID: ; PMCID: . 24459223 PMC3902343

[27] Hussain MM, Arif KS. Bizarre Parosteal Osteochondromatous Proliferation causing angular deformities: A Case Report. J Orthop Case Rep. 2015 Jan-Mar;5(1):45-47. DOI: 10.13107/jocr.2250-0685.253. PMID: ; PMCID: . 27299019 PMC4719352

[28] Mudgal CS, Jupiter JB. Nora’s Lesion: Bizarre parosteal osteochondromatous proliferation. J Hand Surg Am. 1997;22(4):469-471. DOI:10.1016/S0266-7681(97)80269-0

[29] Salna I, Solanki N, Proudman T. Appearances and Evolution of a Recurrent Nora’s Lesion of the Hand. Eplasty. 2019 Jan 24;19:ic5. PMID: ; PMCID: . 30787965 PMC6360910

[30] Gruber G, Giessauf C, Leithner A, Zacherl M, Clar H, Bodo K, Windhager R. Bizarre parosteal osteochondromatous proliferation (Nora lesion): a report of 3 cases and a review of the literature. Can J Surg. 2008 Dec;51(6):486-489. PMID: ; PMCID: . 19057740 PMC2592582

[31] Moretti B, Di Giovanni A, Martino F, Moretti L, Patella S, Patella V. Nora’s lesion. Clinical and therapeutic considerations. Chir Organi Mov. 2008 May;92(1):45-49. DOI: 10.1007/s12306-008-0038-3. Epub 2008 Mar 14. PMID: . 18343981

[32] Helliwell TR, O’Connor MA, Ritchie DA, Feldberg L, Stilwell JH, Jane MJ. Bizarre parosteal osteochondromatous proliferation with cortical invasion. Skeletal Radiol, 30:282-285, 2001. DOI: DOI: 10.1007/s002560100347 11407720

[33] Meneses MF, Unni KK, Swee RG. Bizarre parosteal osteochondromatous proliferation of bone (Nora’s lesion). Am J Surg Pathol. 1993 Jul;17(7):691-697. DOI: 10.1097/00000478-199307000-00006. PMID: . 8317609

[34] Rybak LD, Abramovici L, Kenan S, Posner MA, Bonar F, Steiner GC. Cortico-medullary continuity in bizarre parosteal osteochondromatous proliferation mimicking osteochondroma on imaging. Skeletal Radiol. 2007 Sep;36(9):829-834. DOI: 10.1007/s00256-007-0300-z. Epub 2007 Apr 12. PMID: . 17437102

[35] Zambrano E, Nosé V, Perez-Atayde AR, Gebhardt M, Hresko MT, Kleinman P, Richkind KE, Kozakewich HP. Distinct chromosomal rearrangements in subungual (Dupuytren) exostosis and bizarre parosteal osteochondromatous proliferation (Nora lesion). Am J Surg Pathol. 2004 Aug;28(8):1033-1039. DOI: 10.1097/01.pas.0000126642.61690.d6. PMID: . 15252309

[36] Khamsi B, De la Roza G, Damron T. Forearm mass in a 15-year-old girl. Clin Orthop Relat Res. 2007. 463. 229-236. DOI: 10.1097/BLO.0b013e3180439c1e. 17310927

[37] Öztürk R, Arikan ŞM, Beltir G, Bulut EK, Kekeç AF, Güngör BŞ. Management of Nora’s Lesion: Case Series. *Acta Oncologica Turcica*. 2018. 51(3), 441-445. DOI: 10.5505/aot.2018.37167

[38] Lin CH, Wu K. Nora’s lesion of the distal ulna: a case report. J Int Med Res. 2021 Dec;49(12):3000605211064390. DOI: 10.1177/03000605211064390. PMID: ; PMCID: . 34929099 PMC8721720

[39] Kuruvilla S, Marco R, Raymond AK, Al-Ibraheemi A, Tatevian N. BizarreParosteal Osteochondromatous Proliferation (Nora’s lesion) with translocation t(1;17)(q32;q21): a case report and role of cytogenetic studies on diagnosis. Ann Clin Lab Sci. 2011 Summer;41(3):285-287. PMID: . 22075515

[40] Matsui Y, Funakoshi T, Kobayashi H, Mitsuhashi T, Kamishima T, Iwasaki N. Bizarre parosteal osteochondromatous proliferation (Nora’s lesion) affecting the distal end of the ulna: a case report. BMC Musculoskelet Disord. 2016 Mar 16;17:130. DOI: 10.1186/s12891-016-0981-3. PMID: ; PMCID: . 26984018 PMC4793759

[41] Ting BL, Jupiter JB. Recurrent bizarre parosteal osteochondromatous proliferation of the ulna with erosion of the adjacent radius: case report. J Hand Surg Am. 2013 Dec;38(12):2381-2386. DOI: 10.1016/j.jhsa.2013.09.024. Epub 2013 Nov 1. PMID: . 24183508

[42] Hefferman EJ, Lee CH, Alkubaiden FO, et al. Bizarre parosteal osteochondromatous proliferation of the ulna. Eur J Radiol Extra. 2008;66:e47-e50.

[43] Thacker M, Bugnone A, Humble S, Pitcher J, Scully S. Forearm Mass in an 11-year-old. Clin Orthop Relat Res. 2006. 451. 283-289. DOI: 10.1097/01.blo.0000203482.05959.9f. 16801859

[44] Bush JB, Reith JD, Meyer MS. Bizarre parosteal osteochondromatous proliferation of the proximal humerus: case report. Skeletal Radiol 2007;36:535-540. DOI: 10.1007/s00256-006-0236-8 17492328

[45] Pradhan D, Swain BM, Lenka A, Samal BP. Bizarre parosteal osteochondromatous proliferation of humerus with unusual presentation: a report of one atypical case. *Int Surg J.* 2014. 1, 94-96. DOI: 10.5455/2349-2902.isj20140802

[46] Smith NC, Ellis AM, McCarthy S, McNaught P. Bizarre parosteal osteochondromatous proliferation: a review of 7 cases. *Aust N Z J Surg.* Oct 1996;66(10):694–697. DOI: 10.1111/j.1445-2197.1996.tb00720.x 8855926

[47] Bhalla VK, Coulson H, Parker W, Wynn J, Pipkin WL, Howell CG, Toscano M, et al. Popliteal pseudoaneurysm caused by Nora’s lesion of the femur in a young child: a rare presentation and first report. J Pediatr Surg. 2012;47(12):e55-e59. DOI: 10.1016/j.jpedsurg.2012.09.052 23217920

[48] Takahashi R, Matsuo T, Kawanami K, Takata T, Takahashi E, Deie M. Bizarre parosteal osteochondromatous proliferation of the femur: A case report. *J Orthop.* 2018;15(2):606-609. Published 2018 May 7. DOI: 10.1016/j.jor.2018.05.009 29881204 PMC5990242

[49] Kershen LM, Schucany WG, Gilbert NF. Nora’s lesion: bizarre parosteal osteochondromatous proliferation of the tibia. Proc (Bayl Univ Med Cent). 2012;25(4):369-371. DOI: 10.1080/08998280.2012.11928880 23077391 PMC3448582

[50] Teoh KH, Shortt N, Wilkinson G, Salter DM, Robb JE, Porter DE. Bizarre parosteal osteochondromatous proliferation of the metatarsal: a pediatric case report and archival review. J Foot Ankle Surg. 2009 Nov-Dec;48(6):690.e7-690.e11. DOI: 10.1053/j.jfas.2009.06.012. Epub 2009 Sep 15. PMID: . 19857830

[51] Choi JH, Gu MJ, Kim MJ, Choi WH, Shin DS, Cho KH. Fibrosarcoma in bizarre parosteal osteochondromatous proliferation. Skeletal Radiol. 2001;30(1):44-47. DOI: 10.1007/s002560000265 11289634

[52] Pal JN, Kar M, Hazra S, Basu A. Differential diagnosis of BPOP arising in relation to patella. J Orthop Case Rep. 2015 Oct-Dec;5(4):3-6. DOI: 10.13107/jocr.2250-0685.331. PMID: ; PMCID: . 27299085 PMC4845450

[53] Nilsson M, Domanski HA, Mertens F, Mandahl N. Molecular cytogenetic characterization of recurrent translocation breakpoints in bizarre parosteal osteochondromatous proliferation (Nora’s lesion). Hum Pathol. 2004 Sep;35(9):1063-1069. DOI: 10.1016/j.humpath.2004.02.008. PMID: . 15343507

[54] Rushing CJ, Rogers DE, Spinner SM, Gajzer DC. A Case Report of Heel Pain Mimicking Plantar Fasciitis and Osteosarcoma: A Unique Presentation of a Nora’s Lesion. J Foot Ankle Surg. 2017 May-Jun;56(3):670-673. DOI: 10.1053/j.jfas.2017.01.028. Epub 2017 Mar 3. PMID: . 28268143

[55] Rottler P, Wilke A, Kasper HU, Hütter F. First presentation of a Nora-lesion of the talus in a paraossal fasciitis. Orthop Rev (Pavia). 2019 Mar 27;11(1):7628. DOI: 10.4081/or.2019.7628. PMID: ; PMCID: . 30996837 PMC6452091

[56] Colangeli M, Spinnato P, Zarantonello P, Bendandi B, Donati DM. Nora’s Lesion in a Child: A Case of Complete Spontaneous Regression. Balkan Med J. 2021 Jan;38(1):57-58. DOI: 10.4274/balkanmedj.galenos.2020.2020.7.224. PMID: . 32856884 PMC8909222

[57] Doganavsargil B, Argin M, Sezak M, Kececi B, Pehlivanoglu B, Oztop F. A bizarre parosteal osteochondromatous proliferation (Nora’s lesion) of metatarsus, a histopathological and etiological puzzlement. Joint Bone Spine. 2014 Dec;81(6):537-540. DOI: 10.1016/j.jbspin.2014.07.008. Epub 2014 Sep 22. PMID: . 25245639

[58] Horiguchi H, Sakane M, Matsui M, Wadano Y. Bizarre parosteal osteochondromatous proliferation (Nora’s lesion) of the foot. Pathol Int. 2001 Oct;51(10):816-823. DOI: 10.1046/j.1440-1827.2001.01271.x. PMID: . 11881737

[59] Mahajan S, Chandra R, Mohan Lal Y. “Nora lesion” - Bizarre parosteal osteochondromatous proliferation. J Clin Orthop Trauma. 2012 Dec;3(2):119-121. DOI: 10.1016/j.jcot.2012.07.001. Epub 2012 Jul 27. PMID: ; PMCID: . 26403451 PMC3872810

[60] Suresh S. Nora’s lesion of the second toe. Indian J Orthop. 2010 Jul;44(3):342-344. DOI: 10.4103/0019-5413.65150. PMID: ; PMCID: . 20697492 PMC2911939

[61] Takeda S, Nishimura A, Nakazora S, Sudo A, Hirata H, Kato K. A bizarre parosteal osteochondromatous proliferation at the sesamoid bone of the hallux: A case report. J Orthop Surg (Hong Kong). 2019 Jan-Apr;27(1):2309499019828511. DOI: 10.1177/2309499019828511. PMID: . 30776964

[62] Yao R, Goh EL, Fan Z, Wu X, Feng Y. Bizarre parosteal osteochondromatous proliferation co-occurring with a metatarsal fatigue fracture: a case report. BMC Musculoskelet Disord. 2020 Mar 12;21(1):161. DOI: 10.1186/s12891-020-3168-x. PMID: ; PMCID: . 32164617 PMC7069180

[63] Walsh JC, Murphy D, Freihaut RB, O’Keane JC, Stephens MM. Bizarre parosteal osteochondromatous proliferation of the fifth metatarsal (Nora’s lesion)-Case report. *Foot Ankle Surg.* 2006;12(4):211–214. DOI: 10.1016/j.fas.2006.04.004

[64] Efstathopoulos NE, Papagelopoulos PJ, Lazarettos IT, Savvidou OD, Kaseta MA, Giannakou N, Papachristou GK. Bizarre parosteal osteochondromatous proliferation of the second metatarsal bone (Nora’s lesion). Orthopedics. 2005 Feb;28(2):168-170. DOI: 10.3928/0147-7447-20050201-21. PMID: . 15751373

[65] Harty JA, Kelly P, Niall D, O’Keane JC, Stephens MM. Bizarre parosteal osteochondromatous proliferation (Nora’s lesion) of the sesamoid: a case report. Foot Ankle Int. 2000 May;21(5):408-412. DOI: 10.1177/107110070002100509. PMID: . 10830660

[66] Noguchi M, Ikoma K, Matsumoto N, Nagasawa K. Bizarre parosteal osteochondromatous proliferation of the sesamoid: an unusual hallux valgus deformity. Foot Ankle Int 2004;25:503-506. DOI: 10.1177/107110070402500710 15319109

[67] Bandiera S, Bacchini P, Bertoni F. Bizarre osteochondromatous proliferation of bone. *Skeletal Radiol.* 1998;27:154. DOI: 10.1007/s002560050355 9554006

[68] Endo M, Hasegawa T, Tashiro T, et al. Bizarre parosteal osteochondromatous proliferation with a t(1;17) translocation. *Virchows Arch* 2005;447:99-102. DOI: 10.1007/s00428-005-1266-7 15926071

[69] Mollica AJ, Getz B, Ezike C, Brannick B, Mollica AJ. Nora’s Lesion: Bizarre Parosteal Osteochondromatous Proliferation Causing Splay Foot Deformity: A Case Report. J Am Podiatr Med Assoc. 2019 Nov;109(6):463-466. DOI: 10.7547/17-009. PMID: . 31755776

[70] Prodinger PM, Pilge H, Prantl F, Lauen J. Orthopaedic case of the month: A 16-year-old boy with a recurrent mass of the first toe. Clin Orthop Relat Res. 2011 Apr;469(4):1216-1221. DOI: 10.1007/s11999-011-1787-0. Epub 2011 Jan 29. PMID: ; PMCID: . 21279486 PMC3048266

[71] Nayak AR, Yamsani AK, Pathan AA. Paraosteal osteochondromatosis proliferation (Nora’s lesion) of the great toe. Int J Orthop Sci 2017;3(1):111-113. DOI: 10.22271/ortho.2017.v3.i1b.20

[72] Holmes C, Choksi P, Wrobel JS. Bizarre parosteal osteochondromatous proliferation: a novel case report of recurrence in the toe. J Am Podiatr Med Assoc. 2015;105(1):80–84. DOI: 10.7547/8750-7315-105.1.80. 25675230

[73] James A, Henderson S. Multiple recurrences of subungual exostosis in a child: a unique presentation of a Nora’s lesion. Foot Ankle Int. 2013 Mar;34(3):445-447. DOI: 10.1177/1071100712469336. Epub 2013 Jan 15. PMID: . 23520304

[74] Mohammad A, Kilcoyne A, Blake S, Phelan M. Second toe swelling: Nora’s lesion or glomus tumour, case report and literature review. Ir J Med Sci. 2012 Sep;181(3):357-360. DOI: 10.1007/s11845-009-0435-0. Epub 2009 Oct 8. PMID: . 19813049

[75] Dashti HM, Reith JD, Schlott BJ, Lewis EL, Cohen DM, Bhattacharyya I. Bizarre parosteal osteochondromatous proliferation (Nora’s Lesion) of the mandible. a rare bony lesion. Head Neck Pathol. 2012 Jun;6(2):264-269. DOI: 10.1007/s12105-011-0311-x. Epub 2011 Nov 18. PMID: ; PMCID: . 22094873 PMC3370025

[76] Nobusawa A, Sano T, Negishi A, Yokoo S, Yamaguchi T, Oyama T. Bizarre parosteal osteochondromatous proliferation of the maxilla: a case report. Oral Surg Oral Med Oral Pathol Oral Radiol. 2012 Oct;114(4):e20-e24. DOI: 10.1016/j.oooo.2012.01.025. Epub 2012 May 12. PMID: . 22986254

[77] Kim, S.M., Myoung, H., Lee, S.S. et al. Bizarre parosteal osteochondromatous proliferation in the lingual area of the mandibular body versus osteochondroma at the mandibular condyle. *World J Surg Onc* 14, 35 (2016). DOI: 10.1186/s12957-016-0777-9 PMC4750297 26865041

[78] Baz R, Niscoveanu C. Bizarre Parosteal Osteochondromatous Proliferation of the Skull in a Young Male. Open J Radiol 2013; 3:133-135. DOI: 10.4236/ojrad.2013.33022

[79] Khalele B.A. Nora’s Lesion of The Anterior Maxilla: A rare case report and literature review. *Rev Esp de Patol.* 2016. 49, 19-22. DOI:10.1016/j.patol.2015.11.003

[80] Shakib K, Kalsi H, Tsiridis E, Kumar M. Rare case of bizarre parosteal osteochondromatous proliferation presenting in the zygoma. Br J Oral Maxillofac Surg. 2010. DOI:I:10.1016/j.bjoms.2009.06.157 20719415

[81] Mors M, Cervantes SS, Hinni M. Bizarre parosteal osteochondromatous proliferation presenting in the nasal dorsum. *J Oral Maxillofac Pathol.* 2015;19(1):109. DOI:10.4103/0973-029X.157225 PMC4451655 26097325

[82] Abdu A, Abdulgadir B, Both Sultan A, Arthur A. Spinal Intradural Extramedullary Bizarre Parosteal Osteochondromatous Proliferation of Bone (Nora’s Lesion): First Case Report. *Asian J Neurosurg.* 2019;14(1):304-306. DOI:10.4103/ajns.AJNS_163_18 30937061 PMC6417357

[83] Dorfman HD, Czerniak B. Bone cancers. Cancer. 1995 Jan 1;75(1 Suppl):203-210. DOI: 10.1002/1097-0142(19950101)75:1+<203::aid-cncr2820751308>3.0.co;2-v. PMID: . 8000997

[84] Yuen M, Friedman L, Orr W, Cockshott WP. Proliferative periosteal processes of phalanges: a unitary hypothesis. Skeletal Radiol. 1992;21(5):301-303. DOI: 10.1007/BF00241768. PMID: . 1502582

[85] Ostrowski ML, Spjut HJ. Lesions of the bones of the hands and feet. Am J Surg Pathol. 1997;21:676–690. DOI: 10.1097/00000478-199706000-00008 9199646

[86] Kransdorf M.J. Extraskeletal Osseous and cartilaginous tumors. Imaging of Soft Tissue tumors. 2nd ed. Philadelphia: Lippincott Williams& Wilkins, 2006: 449-452.

[87] Bovee JV, Hogendoorn PCW. Cartilage-forming tumours of bone and soft tissue and their differential diagnosis. Current Diagnostic Pathology 2001 Dec;7(4):223-4. DOI: 10.1054/cdip.2001.0082

